# Interdependence in the therapeutic compliance of hypertensive older adults during the COVID-19 pandemic

**DOI:** 10.1590/1980-220X-REEUSP-2021-0537

**Published:** 2022-03-23

**Authors:** Thamyres de Oliveira Lopes, Jéssica de Castro Santos, Graziele Ribeiro Bitencourt, Angélica Mônica Andrade, Renan Alves Silva, Rafael Oliveira Pitta Lopes

**Affiliations:** 1 Universidade Federal do Rio de Janeiro, Escola de Enfermagem Anna Nery, Rio de Janeiro, RJ, Brazil.; 2 Universidade Federal do Rio de Janeiro, Instituto de Enfermagem, Macaé, RJ, Brazil.; 3 Universidade Federal do Espírito Santo, Centro Universitário do Norte do Espírito Santo, São Mateus, ES, Brazil.

**Keywords:** Adaptation, Treatment Adherence and Compliance, Hypertension, Aged, Coronavirus Infections, Nursing Theory, Adaptación, Cumplimiento y Adherencia al Tratamiento, Hipertensión, Anciano, Infecciones por Coronavirus, Teoría de Enfermería, Adaptação, Cooperação e Adesão ao Tratamento, Hipertensão, Idosos, Infecções Por Coronavírus, Teoria de Enfermagem

## Abstract

**Objective:**

to analyze stimuli and behaviors related to interdependence and their implications for compliance with the therapeutic regimen of older adults with hypertension during the COVID-19 pandemic.

**Method:**

a multiple case, qualitative study, carried out with fifteen older adults treated at a Family Health Strategy unit. A characterization instrument and semi-structured interview were used for data collection. Data were processed in NVivo12, submitted to thematic content analysis, based on Roy’s interdependence mode.

**Results:**

the reports seized showed that the family has meaning as a therapeutic support network, as well as health services, neighbors, friends and religious institutions. Two categories emerged: Stimuli and adaptive behaviors related to interdependence in the pandemic: implications for compliance; Ineffective stimuli and behaviors related to interdependence in the pandemic: implications for compliance.

**Conclusion:**

adaptive and ineffective behaviors related to interdependence during the adjustment to the new condition of social distancing demonstrate the need for greater professional attention to achieve compliance with treatment.

## RESUMO

**Objetivo:**

analisar estímulos e comportamentos relacionados à interdependência e suas implicações para a adesão ao regime terapêutico de idosos com hipertensão arterial sistêmica durante a pandemia de COVID-19.

**Método:**

estudo de casos múltiplos, qualitativo, realizado com quinze idosos atendidos em uma unidade de Estratégia Saúde da Família. Utilizaram-se para coleta de dados instrumento de caracterização e entrevista semiestruturada. Os dados foram tratados em software NVivo12, submetidos à análise de conteúdo temático, a partir do modo de interdependência de Roy.

**Resultados:**

os relatos apreendidos demostraram que a família tem significado enquanto rede de apoio terapêutico, assim como serviços de saúde, vizinhos, amigos e instituições religiosas. Emergiram as categorias: Estímulos e comportamentos adaptativos relacionados à interdependência na pandemia: implicações para adesão; Estímulos e comportamentos ineficazes relacionados à interdependência na pandemia: implicações para adesão.

**Conclusão:**

comportamentos adaptativos e ineficazes relacionados à interdependência durante o ajustamento à nova condição do distanciamento social demonstram a necessidade de maior atenção profissional para o alcance da adesão ao tratamento.

## RESUMEN

**Objetivo:**

analizar estímulos y comportamientos relacionados con la interdependencia y sus implicaciones para la adherencia al régimen terapéutico de ancianos con hipertensión arterial sistémica durante la pandemia de COVID-19.

**Método:**

estudio cualitativo de casos múltiples, realizado con quince ancianos atendidos en una unidad de Estrategia de Salud de la Familia. Para la recolección de datos se utilizó un instrumento de caracterización y una entrevista semiestructurada. Los datos fueron procesados en el software NVivo12, sometidos al análisis de contenido temático, con base en el modo de interdependencia de Roy.

**Resultados:**

los relatos aprehendidos mostraron que la familia tiene sentido como red de apoyo terapéutico, así como los servicios de salud, vecinos, amigos e instituciones religiosas. Surgieron las siguientes categorías: Estímulos y comportamientos adaptativos relacionados con la interdependencia en la pandemia: implicaciones para la adherencia; Estímulos y comportamientos ineficaces relacionados con la interdependencia en la pandemia: implicaciones para la adherencia.

**Conclusión:**

las conductas adaptativas e ineficaces relacionadas con la interdependencia durante el ajuste a la nueva condición de distanciamiento social demuestran la necesidad de una mayor atención profesional para lograr la adherencia al tratamiento.

## INTRODUCTION

Hypertension is a multifactorial clinical condition characterized by persistent elevation of blood pressure levels greater than or equal to 140 and/or 90 mmHg^(1)^. In Brazil and worldwide, it is a serious public health concern and is the main modifiable risk factor for the development of cardiovascular, cerebrovascular, chronic kidney disease and premature death^(2)^.

One of the factors associated with the increased prevalence of hypertension is population aging^(3)^. It is estimated that the elderly population accounts for approximately 17% of the total population of Brazil^(4)^. There is a observed prevalence of approximately 65% of hypertension in this subgroup, contributing directly or indirectly to 45% of deaths from cardiovascular disease^(1)^.

The therapeutic approach to hypertension in older adults involves strategies aimed at controlling blood pressure levels and secondary damage, either by various pharmacological measures or by non-pharmacological measures, based on the change in harmful behaviors and/or lifestyle^(1)^. Successful antihypertensive treatment depends on complying with these measures. Studies developed with older adults indicate that there are contributing factors to a greater difficulty in complying with therapeutic measures, such as reduced cognitive capacity, hearing or visual loss, loss of dexterity, depression, certain health beliefs and the lack of accessibility to drugs^(5)^ as well as a relationship between the frailty syndrome of older adults and non-compliance^(6)^.

Peculiarities of the aging process and attitudes that involve changes in life habits go beyond the exclusivity of the self, reaching the social self and its relationships. Thus, the therapeutic approach requires the selection of several strategies that make achieving treatment compliance a challenge as well as highlight the need for the inclusion of a support network.

A support network is an important ally in hypertension treatment, facilitating the process of complying and encouraging self-care practices^(7)^. Within the disciplinary perspective of nursing, especially for Roy’s Adaptation Model, interdependence is the adaptive mode responsible for responding to stimuli about close relationships between people and their support systems as well as affective needs such as affection and love^(8)^.

Despite the recognized relevance of a support network and affections for hypertension treatment in older adults, initiatives are still incipient to investigate the perceptions of this group. In addition to the lack of investigations, the occurrence of social isolation, discrimination, violence and the abandonment of this group by its support network^(9)^ stand out. Associated with these challenges, new implications have been observed since the COVID-19 pandemic was declared by the World Health Organization. It was observed that people over 60 years of age are more vulnerable to worse outcomes of the disease such as hospitalization, tracheal intubation, mechanical ventilation, hospitalization time, and death^(10)^. Furthermore, in addition to the great threat to life, the pandemic can put the most at risk of poverty, loss of social support, trauma of stigma, discrimination, and isolation^(11)^.

In this regard, investigations on the perceptions of interdependence can contribute to developing strategies that aim at the joint participation of the family, support network and where the mechanisms of affectivity are valued for therapeutic compliance during the COVID-19 pandemic. Considering the above, it was proposed as a guide question of this investigation: how do older independent adults recognize the support network, affections, affection and love to comply with the therapeutic regimen of hypertension during the COVID-19 pandemic? This investigation aimed to analyze stimuli and behaviors related to interdependence and its implications for the treatment regimen of older adults with hypertension during the COVID-19 pandemic.

## METHOD

### Study Design and Location

This is a multiple case, exploratory descriptive, qualitative study. It was developed at a health unit in the Family Health Strategy model in a municipality in northern Rio de Janeiro State, Brazil. The choice was made by this unit to meet a high number of older adults living with hypertension in the municipality.

### Population and Sample Definition

Convenience sampling was composed of fifteen independent older adults, of both sexes, with medical diagnosis of hypertension under treatment, assisted in the unit, defined by the criterion of empirical and theoretical saturation arising from the absence of new themes and sufficient conceptual depth to understand and relate the emerging theoretical categories^(12)^.

### Inclusion and Exclusion Criteria

Independent older adults who obtained independence for all functions considered for activities of daily living determined by the Katz Index^(13)^ were included. Older adults who had an inability to maintain dialogue, understanding and/or verbalization and with a history of senile disease or cognitive deterioration were excluded from the study.

The ambience, recruitment of participants and data collection process occurred during activities related to the actions by the vaccination campaign for COVID-19, carried out in the unit selected for the research. Participants were invited directly to integrate the study, based on general information about the research and the study method, and thus directed to a consultation room, in the unit itself, so that privacy was maintained during data collection. No older adults recruited were dependent on the Katz Index and ten older adults refused to participate in the survey because they did not have time availability.

### Data Collection

Data were collected between February and April 2021 by the mean researcher. This is a nursing academic with previous training by the guiding professor in data collection procedures and with academic experience in research groups.

Data collection was developed in two stages, with an average duration of forty minutes, consisting of a meeting with each participant. The first stage was the application of a sociodemographic characterization instrument, based on sex, age, education, marital status, religion, income, comorbidities and mean time of hypertension diagnosis variables.

The second stage occurred through an interview guided by a semi-structured script with open-ended questions related to their affections, family relationships, support network and their contributions to therapeutic compliance. The interviews’ audio was recorded on MP4 digital equipment. The average interview time was thirty minutes.

### Data Analysis and Treatment

Sociodemographic characterization was analyzed with descriptive statistics, using the calculations of mean and percentage. The data from the interviews were transcribed and processed in NVivo12, submitted to cluster analysis, forming clusters according to word similarities. To perform data analysis and interpretation, category thematic content analysis was used following the following steps: 1) pre-analysis; 2) material exploitation; 3) treatment of results, inference and interpretation^(14)^. Due to the sanitary limitations imposed by the pandemic, the participants were not returned to the interviews’ transcripts. The analysis of thematic category content made it possible to arrange the units of analysis and registration through categories that were composed using the corpora contained in the nodes and the deduction of Roy’s Adaptation Model elements. This process aimed to insure the theoretical density through sequential clusters to obtain the thematic categories. The theoretical densification was proven by Pearson’s correlation ≥0.60^(15)^. Then, the categories were validated by two researchers from the research team with knowledge based on the theoretical model from their experiences of application in care, research and teaching.

### Ethical Aspects

To carry out the research, all ethical requirements were respected, as recommended by Resolution 466/2012 of the Brazilian National Health Council (*Conselho Nacional de Saúde*). The project was approved under Opinion 4,495,822, dated January 2021. All participants accepted and signed the Informed Consent Form (ICF). In order to guarantee anonymity, the research participants were identified by alphanumeric codes, with the letter P, followed by the sequence number of the interviews.

## RESULTS

The study included 15 independent older adults living with hypertension, 53.3% of which were women, aged between 60 and 78 years, mean age of 69 years; 40% attended incomplete elementary school; 46.7% were married, while 26.7% were widowed; 60% reported receiving up to one minimum wage; 93.3% claim to have some religious inclination. Associated with hypertension, 40% reported having diabetes mellitus, and 33.3% reported having heart disease. The mean time of diagnosis and onset of hypertension treatment were similar, 20 years (40%), followed by 10 years (33.3%).

**Figure 1.**
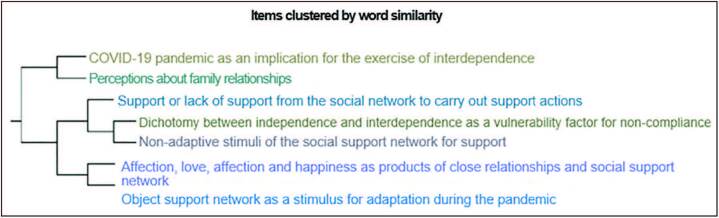
Cluster analysis dendrogram applied to the interviewees’ speeches, Macaé – RJ, Brazil, 2021.

Source: Nvivo12.

From word similarity, interrelated clusters emerged forming a dendrogram expressed in Figure 1. Two thematic categories emerged, formed from analysis and insertion of cluster data.

### Stimuli and Adaptive Behaviors Related to Interdependence in the Pandemic: Implications for Compliance

It addresses four themes, represented by the thematic nodes: affection, love, affection and happiness as products of close relationships and social support network; object support network as a stimulus for adaptation during the pandemic; support or lack of support from the social network to carry out support actions; and perceptions about family relationships.

Regarding the perceptions about the contributions of close relationships and the support network for carrying out the necessary adaptations, coping with hypertension and compliance with treatment, there were indications in the speeches of the existence of a network comprising family members, a health care network and an object support network. Thus, both stimuli and behaviors favorable to adaptation and adaptation were evidenced. Aspects emerged by the participants as fundamental for continuity of hypertension treatment, demonstrating that, when they find support, they can improve their health condition.

*Only my daughter right, who is helping me a lot in this regard. Because sometimes I get a little forgotten, right, then she keeps reminding me, guiding me and we see many cases happening, then we see that it is not feeling very well and that is something that sometimes has to correct right. Is this experience good, right? At least I’m trying to do different, trying to decrease salt, sugar, oil, these things. (P5)*

*I, knowing that I am hypertensive, I need these people (health professionals), otherwise I will be without people to guide me, so I know what to do, where to go, I will be without help, without knowing where to ask for help, because I will be disoriented, disoriented, there is no other way. (P3)*

*But I try my best not to get bored, sometimes I’m at home and i look for a little something to entertain to do. Sometimes, there’s nothing, I like some plants, I handle the plants to help with my survival and also my pressure so it doesn’t go up, because it’s out of control. (P3)*

In addition to the perceptions based on support for compliance actions, positive discourses related to interdependence were also observed. It was demonstrated that, due to social and religious relations, feelings of affection, happiness, love and affection are processed, decisive in coping with the disease and for health promotion.

*Yeah, so I think feeling loved and wanted when you’re around your family giving you attention, giving you affection, giving you love, you feel that concern for them, for your health, you know, you want to take it with them, so that’s what’s there. (P6)*

*Happiness is to believe that Jesus exists, for me, right, Jesus exists and that you are praying and asking Him, not by wanting more, but you always asking Him for health. (…) I talk to Jesus, to me and all the brothers who meet like me, with some illness in the body, and then I thank God very much that, for sure, my daughter happens. (P6)*

Still from the perspective of close relationships between people and their support systems, it was noticed that, given the stimulus of the COVID-19 pandemic, participants used technological resources to ensure social exchanges and adapted spaces for social interaction, directly implying interdependence. Thus, adaptive responses are observed in the statements in the face of the need for social distancing.

*Oh, and the day I have a dance for myself? (…) then, today, I’m going to dance, then I take things out of the middle of the room, because my room is big, two rooms like this more or less, then I take the sofa, my rocking chair, I leave that half free. and I turn on the stereo and I’m going to dance the bolero, I’m going to dance the waltz and I put on high heels, because I always wear high heels. (P6)*

*There are people who come from Ajuda de Baixo, who I made friends there to spend an afternoon with me. Lately, that hasn’t been happening; lately, we talk a lot by phone on WhatsApp, we talk, looking almost directly, like, at the person, but people come from there and people who are not mine at all, affection and attention, right? (P6)*

*I just live on the phone talking to others, girl, with my friends. I do not leave the house no (…). Then I say to girl, now corona, then I say “go home no, come here not because of corona”. And I have a granddaughter who likes me very much, so she doesn’t leave me at all, who is the daughter of my daughter, of my daughter, understand? And she calls. (P8)*

### Ineffective Stimuli and Behaviors Related to Interdependence in the Pandemic: Implications for Compliance

Four themes are understood: dichotomy between independence and interdependence as a vulnerability factor for non- compliance; non-adaptive stimuli from the social support network for compliance; COVID-19 pandemic as an implication for the exercise of interdependence; and perceptions of family relationships.

Regarding the influence of close relationships and the support network for the actions demanded in hypertension treatment, in the perceptions of some participants, notes regarding its inefficiency are observed. There are stimuli that oppose the treatment regimen coming from the family network, as well as from the health care network in the municipal context.

*Just like I went to my mother, father and sister’s house, and there they had to remove all the salt, because they had a serious blood pressure problem, then my mother would make food without salt and I got used to it, but then I got married, and my husband liked it a lot with salt and he was always complaining that he was out of salt, that I took all the salt out of the dried meat, then, because of him, I lost control. Because I already stayed used to eating without salt practically and with little sweet, because in my mother’s house, it was like this, then I need to decrease, I need to. (P5)*

*Here, in Macaé, there is nothing for older adults, what is in Macaé for older adults? I see dirt courts and other courts there and there, but I don’t see a gym for anyone, I don’t see anything for older adults in each neighborhood, because it has to be, I mean, it has to be, but there isn’t, so you have the physical therapist to follow up, the nursing technician, because you won’t do gymnastics without measuring the pressure, I don’t see that here. (P6)*

Also on the support network, negative processes were identified regarding the possibility of these close relationships becoming absent. Feelings of sadness, hopelessness and abandonment by older adults can be seen in the speeches, when there is a family distance and low commitment on the part of this network due to hypertension demands, which can be considered ineffective stimuli and behaviors for coping with the disease.

*Oh, lost! Lost, not knowing what to do, because without these people and without these places, I will be lost without knowing what to do, who and where to look for help. (P3)*

*Oh, I’d feel bad, I’d feel really bad, feel like I’ve been abandoned. (…) because it’s bad for a person to feel alone. (P4)*

Added to the possibility of lack of affection and the support of the committed support network, it was evidenced in the speeches that, in the face of the COVID-19 pandemic, interdependence was affected. Ineffective behavior stemming from pandemic stimulation was implied. The practice of protective measures, such as social distancing, mask use and environmental control, caused isolation and decreased interaction with support systems.

*Because I live alone and my house is always full (…) now, at this moment of this problem, we are not almost visiting anyone, right? It’s over the phone and, when he arrives at our house, I say “Oh, take off your shoes and put your foot on the carpet, put on the mask and put the alcohol in your hand to enter here in my house”. (P11)*

*No, I’m not going anywhere, I don’t talk to anyone, because, also, with this pandemic, I can’t keep going places, right, staying indoors even for security reasons, then there is no one to talk to. (P12)*

*I’m just waiting for the vaccine now so I can go, because I have to mess with my medication, because there’s some medicine that the doctor thinks I have to mess with (…) yeah, then she gave me a referral for me to go look for it, because I’ve been gone for a long time, right? Because before, it was in the shack, now be there in Dona Alba, but I’m already with the referral, but it’s not scoring because of the pandemic, so I’m waiting now to do the exams. (P2)*

Moreover, it is observed that the state of older adults’ independence and functional capacity can also affect interdependence. The speeches expose behaviors of affirmation of freedom and autonomy, when the necessary actions for hypertension treatment are performed. Consequently, it results in a distancing from its support network and the inefficient exercise of affects, caused by participants voluntarily, even when the network wishes to become present.

*I change my diet myself, I change things, alone, without talking to anyone, because it’s better for a person to do things alone than talking to one and the other. (P4)*

*No, because sometimes she asks, “mom, have you had your checkup yet?” then I say I’ll do and I’ll do, stay there, because I, also, so, I do not like to bother anyone, I do not like to bother anyone. For example, my daughter says, “mom, when you want to shop, tell me, I come here to take you shopping.” I say, “ok”, but now he asks me if I tell someone to take me to the butcher shop, or go to a fair, no, I don’t like to bother anyone, I go there and do it, when I can, when I want, I go there and do. (P6)*

*One day, the person does things for you well, tomorrow, they do well, the day after tomorrow, they do well, and then they don’t want to anymore. So, depending on others, we don’t… have to do everything not to depend on others. Depending on others is very bad. It’s bad for us and it’s bad for the person, because the person is doing it from there, but they’re not… they’re doing it, but they don’t like doing it. Do you understand? So, it’s too bad. (P8)*

## DISCUSSION

Sociodemographic data showed similarities with other studies that prove a higher frequency of diagnosis of hypertension among individuals with a lower level of education^(16)^. Furthermore, it is understood that low education can promote difficulty in understanding the guidelines for therapeutic compliance, especially in illiteracy, as it is directly related to obtaining, understanding and using favorable information to promote their health^(17)^.

It is known that the level of education assessment is related to the socioeconomic level, characterizing higher rates of hypertensive diseases in the population with lower income^(18)^. In this survey, the majority reported receiving up to one minimum wage. Thus, it is understood that socioeconomic differences play an important role in health conditions, through the scope of the problem, access to health systems and compliance with treatment^(19)^.

A study carried out with a profile similar to this study found that married people, when compared to single people, were twice as likely to perform treatment actions properly^(20)^, due to family involvement as a facilitating component for compliance through emotional support in times of difficulty^(7)^. However, it was observed that widowed older adults are influenced by their health and quality of life and therapeutic compliance, because this state can make these people, after years of living with their spouses, considered a significant person, face moments of loneliness in the coming years of grief^(21)^.

In the context of the COVID-19 pandemic, it was observed in the speeches, the adoption of precautionary behaviors, such as social distancing and protection measures, highlighting the response for maintenance and transformation of the environment, favoring their health status^(8)^. Such measures provided a direct impact on the sociability of these older adults and on the performance of practices necessary for continuity of treatment. It is believed that such an impact is motivated by a greater degree of compliance with protective measures for people in greater vulnerability, as they understand their risks of infection and commitment in the face of the new context^(22)^.

However, as seen in some speeches, not all older adults were able to adapt to the stimulus of the pandemic, reporting isolation and impossibility of medication adjustments. Therefore, it is recognized that social isolation can become a source of difficulties in controlling the diseases of this population, increasing the need to monitor their health situation^(23)^. Thus, the supervision of pharmacological treatment, through dose adjustments, when necessary, implies greater regularity in treatment^(24)^. Other studies carried out in the pandemic context report that social isolation in this period has become a reinforcing factor of greater vulnerability, suffering, fear, panic, and concern in older adults^(25,26,27)^.

Thus, these changes affect the subject in its entirety and directly in their interpersonal relationships, harming their interaction with support networks and the benefits reported by them, such as affection, love, affection and happiness. In an attempt to adapt to the new situation, the speeches showed the adequacy of spaces and the use of technologies during the pandemic, in order to guarantee social relations and reduce the impact on interdependence. These findings are supported by Roy’s Adaptation Model, based on the process of awareness and meaning, for the integration between the person and the environment. Therefore, awareness of oneself and one’s environment is rooted in thought and feeling. The change or continuity of a certain behavior arises from the integration of creative processes, resulting in positive or destructive behaviors to their health^(8)^. Thus, the viability of maintaining support networks, based on digital tools, was a way of adapting to social deprivation^(28,29)^.

It was observed that the distance from the support network is also influenced by the physical state of independence of these older adults, with speeches exposing the desire to maintain their autonomy and how the interference of their support systems can influence their freedom to make decisions during treatment actions.

Independence in older adults is one of the first steps towards achieving active aging and a better quality of life, being understood as the ability to carry out activities of daily living through autonomy^(30)^. Despite the recognition of the beneficial factors of independence, participants’ statements point to a social search for autonomy and freedom, causing ineffective behaviors related to interdependence. Understanding that compliance with therapy is a complex phenomenon, requiring a comprehensive understanding of individuals and their affective and social needs, it is believed that it is necessary to recognize the relevance of independence for active aging; however, it is also essential to highlight the possible impediments generated in the affective and social dimension in this population. These elements are considered essential for adaptation to the chronic condition and compliance with treatment.

The study limitations are as follows: the non-return of interview transcripts for validation of participants, due to current health recommendations; the subjectivity employed, despite the maintenance of the researcher’s impartiality, as the study depends on its interpretation; and the absence of other qualitative studies exploring interdependence and compliance in older adults with hypertension. It was found that studies related to Roy’s Theory in hypertensive older adults are little explored. Thus, this study may contribute to clarifying the importance of affections and the support network of older adults in the process of coping with and adapting to hypertension, especially in the context of the pandemic. Thus, the modulation of strategic actions for health promotion and medication compliance will be allowed in this context.

## CONCLUSION

Study participants revealed important issues experienced regarding the mode of interdependence. Some showed ineffective stimuli and behaviors related to interdependence, directly affecting compliance with hypertension treatment and health promotion. Regarding the stimulus of the COVID-19 pandemic, some participants showed ineffective behaviors during the adjustment to the new condition of social distancing, demonstrating the need for greater attention to reach satisfactory adaptation.

Thus, the construction of assistance strategies aligned with older adults’ social and emotional support specificities is relevant. In this sense, the integral approach must be reinforced as a health practice, emphasizing the importance of nurses for the recognition of stimuli and behaviors, aiming at providing assistance that encourages coping mechanisms to achieve adaptation and compliance.

It is recognized the importance of using technologies to achieve the benefits and processes of confrontation arising from the interdependence mode. Through them, older adults can receive affection, affection and emotional support, especially in the context of the COVID-19 pandemic. Even so, the indispensable participation of family, community relations, health systems, social agencies, the network of objects and friendships for compliance and safety actions to hypertension treatment is highlighted.

## ASSOCIATE EDITOR

Cristina Lavareda Baixinho
